# Supplementary material for: Quality assessment of tissue samples stored in a specialized human lung biobank
Source: PLoS One. 2019 Apr 4;14(4):e0203977. doi: 10.1371/journal.pone.0203977 (PMC6448820; doi:10.1371/journal.pone.0203977)
Supplement: S1 Fig — (PDF) [file pone.0203977.s001.pdf]

HE stains of samples shock frozen in liquid N_2 or preserved in RNAlater

Patient 1: male, age: 74 years, Large cell carcinoma, Stage IB

Patient2: male, age: 73 years, Adenocarcinoma, Stage IIB

A: shock frozen in liquid N_2

Patient 1

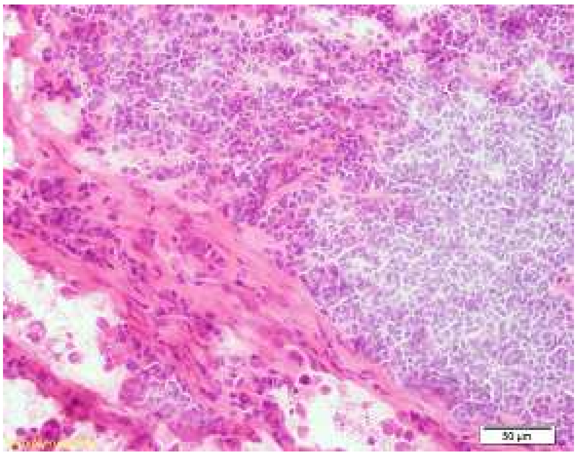

Patient 2

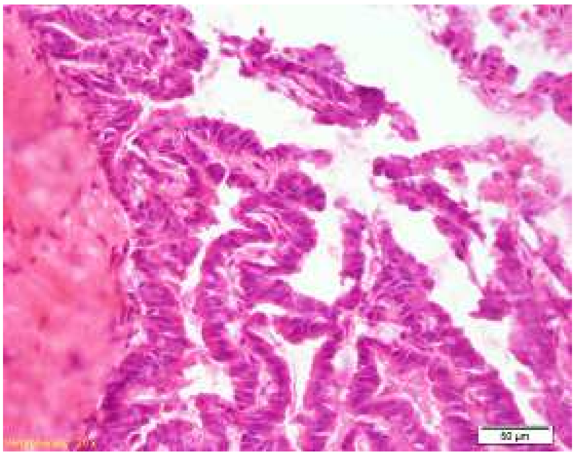

B: RNAlater, 1 d

Patient 1

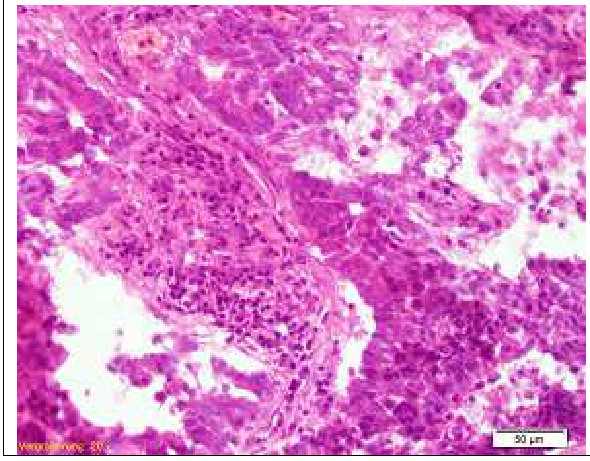

Patient 2

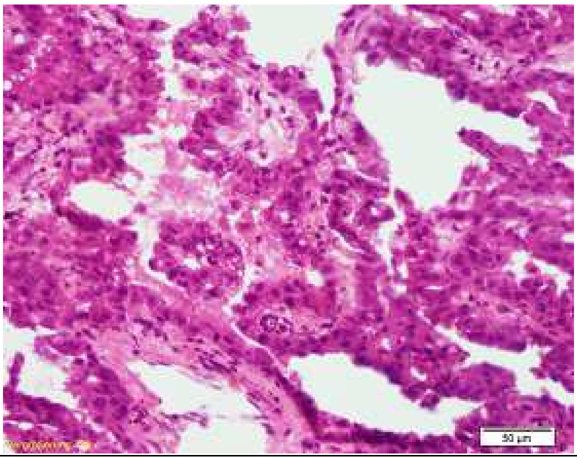

C: RNAlater, 7d d

Patient 1	Patient 2
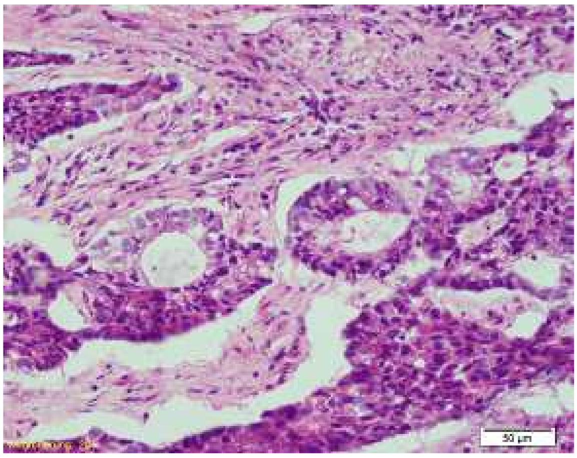	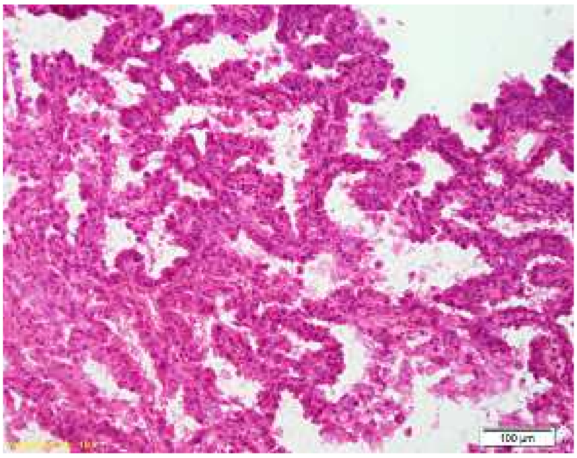
