# Supplementary material for: Quality assessment of tissue samples stored in a specialized human lung biobank
Source: PLoS One. 2019 Apr 4;14(4):e0203977. doi: 10.1371/journal.pone.0203977 (PMC6448820; doi:10.1371/journal.pone.0203977)
Supplement: S1 File — (PDF) [file pone.0203977.s002.pdf]

Absolute expression levels for individual genes and patients for different asservation methods and times

Expression was measured by qRT-PCR in four individual patients. Samples were either fresh frozen in liquid nitrogen (blue), or preserved in RNAlater for 1 day (red) or 7 days (green) before long term storage at -80 °C.

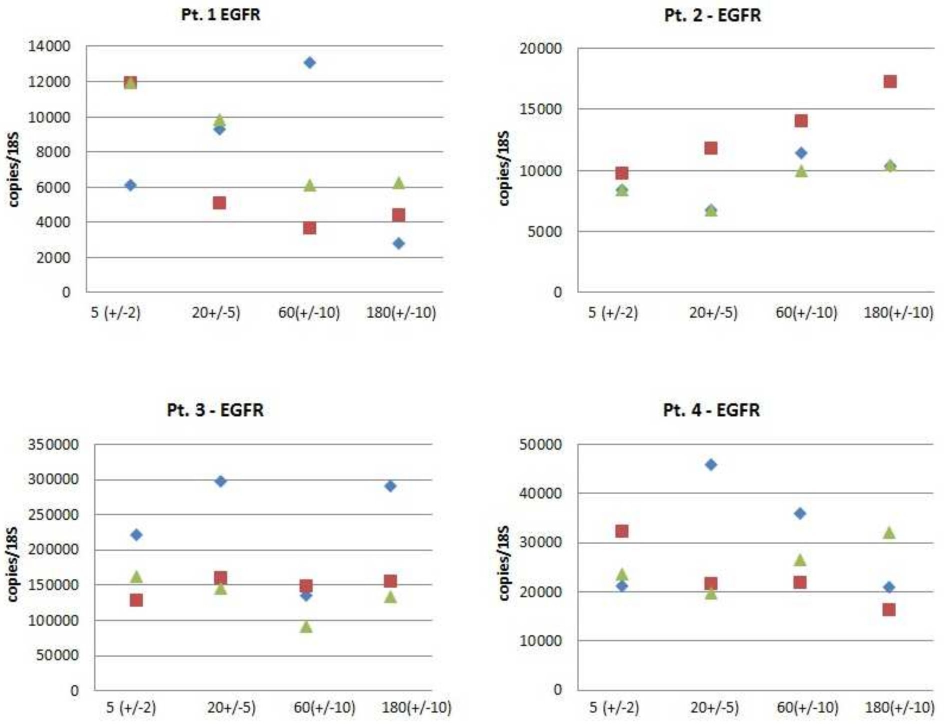

Pt 1, EGFR	N_2	RNAlater 1d	RNAlater 7d
5(+/-2)	6097	11905	11905
20(+/-5)	9291	5109	9821
60(+/-10)	13072	3678	6114
180(+/-10)	2761	4417	6229

Pt 2, EGFR	N_2	RNAlater 1d	RNAlater 7d
5(+/-2)	8425	9774	8383
20(+/-5)	6717	11868	6767
60(+/-10)	11485	14052	10000
180(+/-10)	10408	17267	10464

Pt 3, EGFR	N_2	RNAlater 1d	RNAlater 7d
5(+/-2)	222544	129020	163306
20(+/-5)	297648	161134	145367
60(+/-10)	136025	148534	91763
180(+/-10)	291597	155756	133333

Pt 4, EGFR	N_2	RNAlater 1d	RNAlater 7d
5(+/-2)	21209	32341	23570
20(+/-5)	45959	21783	19808
60(+/-10)	36041	22066	26641
180(+/-10)	21060	16349	32189

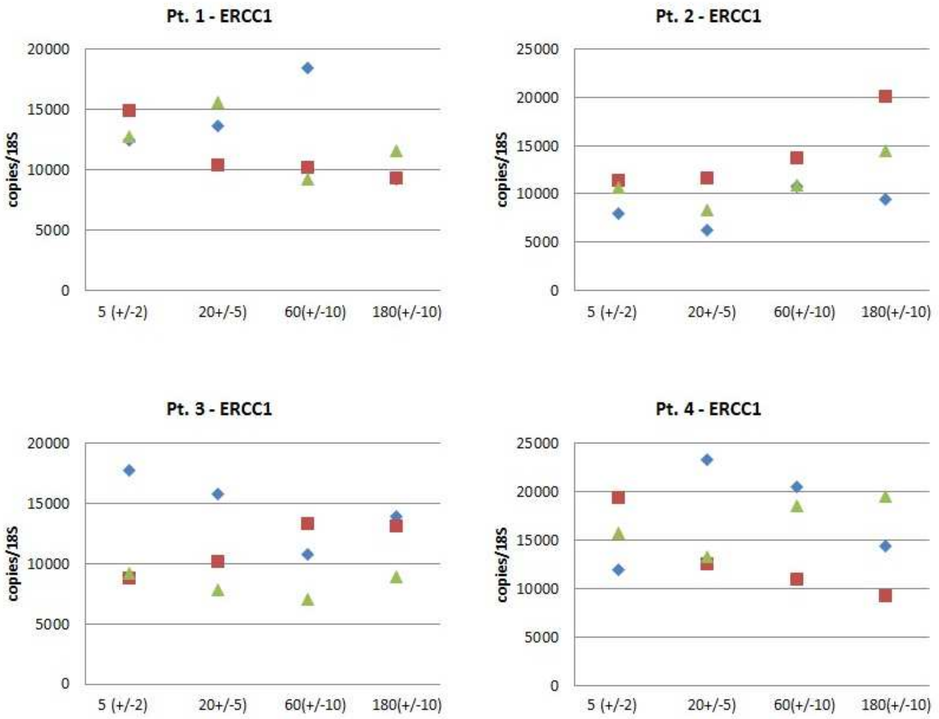

Pt 1, ERCC1	N_2	RNAlater 1d	RNAlater 7d
5(+/-2)	12416	14882	12768
20(+/-5)	13682	10370	15640
60(+/-10)	18488	10248	9263
180(+/-10)	9201	9292	11606

Pt 2, ERCC1	N_2	RNAlater 1d	RNAlater 7d
5(+/-2)	7957	11412	10637
20(+/-5)	6271	11701	8300
60(+/-10)	10742	13786	10915
180(+/-10)	9469	20088	14436

Pt 3, ERCC1	N_2	RNAlater 1d	RNAlater 7d
5(+/-2)	17792	8826	9183
20(+/-5)	15760	10227	7812
60(+/-10)	10839	13352	7066
180(+/-10)	13956	13172	8932

Pt 4, ERCC1	N_2	RNAlater 1d	RNAlater 7d
5(+/-2)	11970	19404	15754
20(+/-5)	23268	12565	13328
60(+/-10)	20486	11012	18594
180(+/-10)	14413	9338	19483

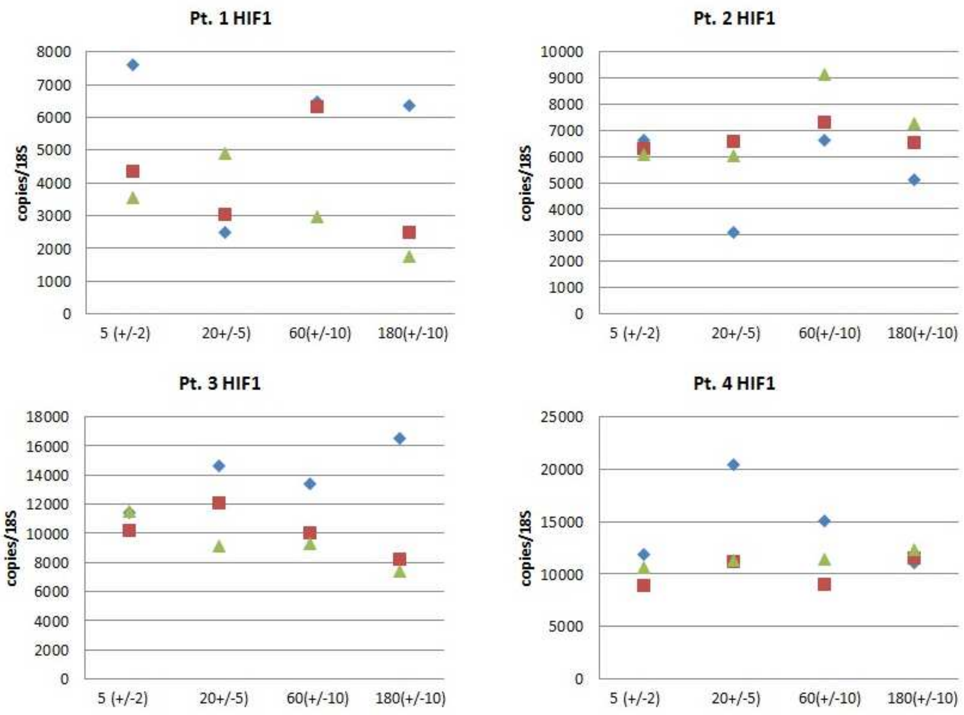

Pt 1, HIF1	N_2	RNAlater 1d	RNAlater 7d
5(+/-2)	7588	4361	3545
20(+/-5)	2494	3043	4896
60(+/-10)	6470	6316	2961
180(+/-10)	6371	2500	1741

Pt 2, HIF1	N_2	RNAlater 1d	RNAlater 7d
5(+/-2)	6634	6322	6068
20(+/-5)	3090	6584	6034
60(+/-10)	6631	7324	9159
180(+/-10)	5113	6553	7284

Pt 3, HIF1	N_2	RNAlater 1d	RNAlater 7d
5(+/-2)	11466	10172	11522
20(+/-5)	14678	12117	9104
60(+/-10)	13399	10000	9324
180(+/-10)	16573	8248	7373

Pt 4, HIF1	N_2	RNAlater 1d	RNAlater 7d
5(+/-2)	11846	8947	10587
20(+/-5)	20475	11148	11314
60(+/-10)	15098	9060	11466
180(+/-10)	11126	11522	12340

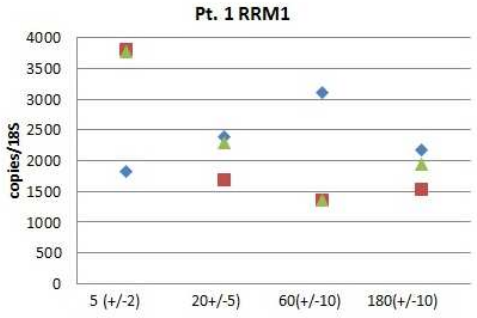

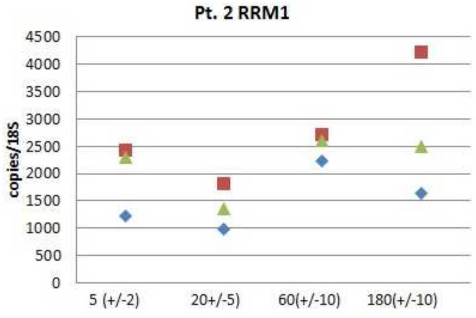

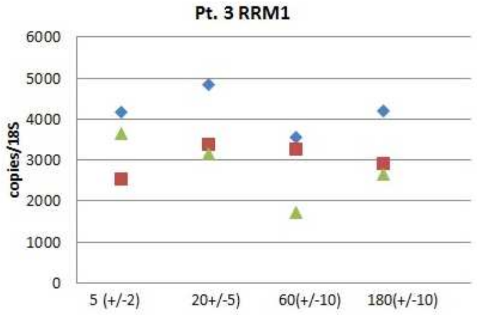

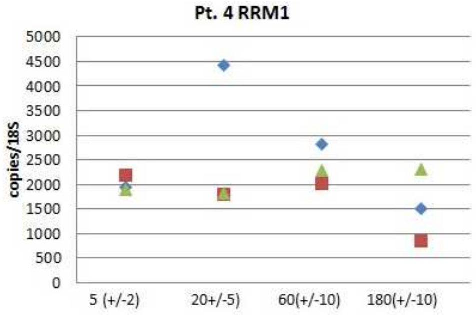

Pt 1, RRM1	N_2	RNAlater 1d	RNAlater 7d
5(+/-2)	1823	3803	3765
20(+/-5)	2397	1698	2304
60(+/-10)	3105	1355	1353
180(+/-10)	2184	1538	1947

Pt 2, RRM1	N_2	RNAlater 1d	RNAlater 7d
5(+/-2)	1215	2434	2304
20(+/-5)	993	1824	1362
60(+/-10)	2233	2717	2593
180(+/-10)	1643	4217	2485

Pt 3, RRM1	N_2	RNAlater 1d	RNAlater 7d
5(+/-2)	4181	2530	3658
20(+/-5)	4840	3393	3153
60(+/-10)	3565	3271	1712
180(+/-10)	4187	2929	2664

Pt 4, RRM1	N_2	RNAlater 1d	RNAlater 7d
5(+/-2)	1943	2198	1901
20(+/-5)	4413	1791	1813
60(+/-10)	2814	2010	2291
180(+/-10)	1497	837	2303

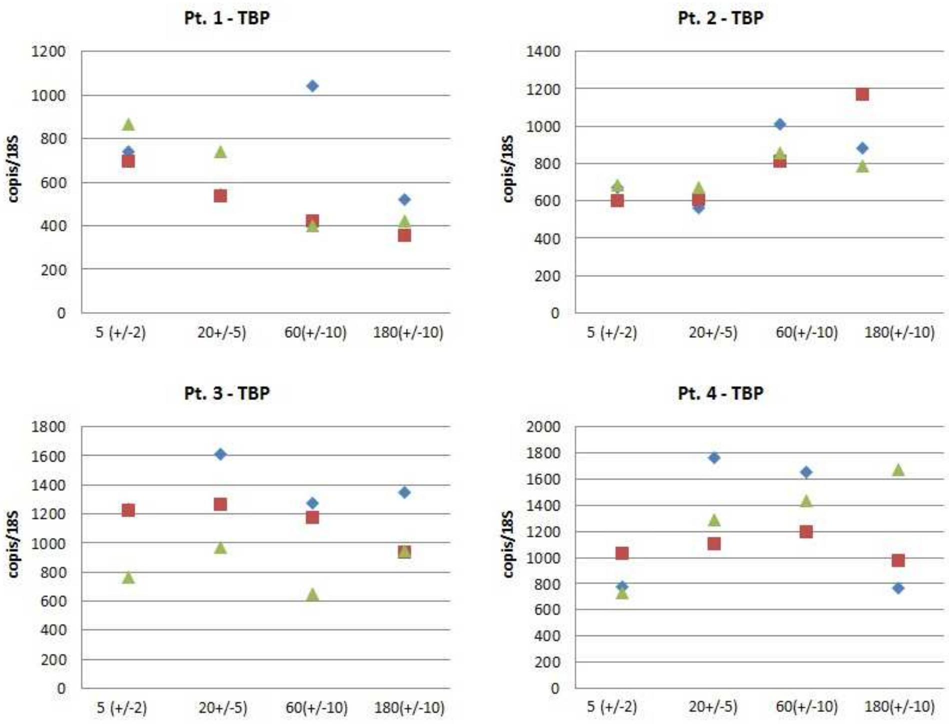

Pt 1, TBP	N_2	RNAlater 1d	RNAlater 7d
5(+/-2)	742	699	865
20(+/-5)	543	537	740
60(+/-10)	1042	420	402
180(+/-10)	521	357	422

Pt 2, TBP	N_2	RNAlater 1d	RNAlater 7d
5(+/-2)	673	599	683
20(+/-5)	566	608	670
60(+/-10)	1014	810	860
180(+/-10)	884	1172	787

Pt 3, TBP	N_2	RNAlater 1d	RNAlater 7d
5(+/-2)	1232	1223	762
20(+/-5)	1614	1270	974
60(+/-10)	1273	1180	649
180(+/-10)	1350	936	948

Pt 4, TBP	N_2	RNAlater 1d	RNAlater 7d
5(+/-2)	781	1028	728
20(+/-5)	1765	1101	1291
60(+/-10)	1650	1199	1435
180(+/-10)	767	980	1676
