# Supplementary material for: Quality assessment of tissue samples stored in a specialized human lung biobank
Source: PLoS One. 2019 Apr 4;14(4):e0203977. doi: 10.1371/journal.pone.0203977 (PMC6448820; doi:10.1371/journal.pone.0203977)
Supplement: S1 Table — (PDF) [file pone.0203977.s003.pdf]

Influence of cold ischemic time and asservation procedure on RNA quality

Overall RNA quality was assessed on an Agilent BioAnalyser. RIN (RNA integrity numbers) for different ischemic times and different asservation methods are given below. Values were used to build Fig.3.

Patient 1	fl. N2	RNAlater 1d	RNAlater 7D
5(+/-2)	8,7	8,9	9,2
20(+/-5)	8,8	7,2	8
60(+/-10)	8	9	7,1
180(+/-10)	9,1	7,7	7,9

Patient 2	fl. N2	RNAlater 1d	RNAlater 7D
5(+/-2)	9,4	8,6	8,4
20(+/-5)	6,5	7,9	9,2
60(+/-10)	8,8	8,8	9
180(+/-10)	8,8	9,2	9,4

Patient 3	fl. N2	RNAlater 1d	RNAlater 7D
5(+/-2)	10	10	8
20(+/-5)	10	9,9	9,8
60(+/-10)	9,5	10	9,8
180(+/-10)	9,8	9,8	9,7

Patient 4	fl. N2	RNAlater 1d	RNAlater 7D
5(+/-2)	9,6	9,9	9,9
20(+/-5)	8,8	9,9	9,5
60(+/-10)	10	9,8	9,7
180(+/-10)	8,9	9,7	9,7
