# Supplementary material for: Quality assessment of tissue samples stored in a specialized human lung biobank
Source: PLoS One. 2019 Apr 4;14(4):e0203977. doi: 10.1371/journal.pone.0203977 (PMC6448820; doi:10.1371/journal.pone.0203977)
Supplement: S2 Table — (PDF) [file pone.0203977.s004.pdf]

RNA quality after thawing and refreezing

Samples had been thawed for more than 24 h and were refrozen before extraction of nucleic acids and analysis. The table S1 demonstrates RIN values as indicator of overall RNA quality measured before thawing and after thawing/refreezing. The tables show amplification curves of realtime-RT-PCR experiments conducted under both conditions for 18S and RRM1. The results indicate that intact RNA could be isolated from samples preserved in RNAlater, whereas the RNA in sample snap frozen in liquid N_2 was degraded.

RIN values

Sample	A	B
Patient 1, shock frozen in liquid N_2	2,8	10
Patient 1, RNAlater 7 d	9,3	9,8
Patient 2, shock frozen in liquid N_2	5,1	8,8
Patient 2, RNAlater 7 d	9,8	9,5

A: after thawing and refreezing; B: without thawing

Realtime-RT-PCR 18S

	A	B
Patient 1, shock frozen in liquid N_2	n.d.	14,71
Patient 1, RNAlater 7 d	14,6	14,79
Patient 2, shock frozen in liquid N_2	n.d.	15
Patient 2, RNAlater 7 d	14,47	15,23

A: after thawing and refreezing; B: without thawing

Realtime-RT-PCR RRM1

	A	B
Patient 1, shock frozen in liquid N_2	n.d.	22,3
Patient 1, RNAlater 7 d	23,4	22,63
Patient 2, shock frozen in liquid N_2	n.d.	23,54
Patient 2, RNAlater 7 d	23,95	24,09

A: after thawing and refreezing; B: without thawing
